# Immunothrombosis in hospitalized COVID-19 patients identified by multiomics profiling and linked to postacute complications

**DOI:** 10.1016/j.isci.2026.116326

**Published:** 2026-06-11

**Authors:** Laura Ansone, Līva Pelcmane, Monta Brīvība, Rihards Saksis, Ivars Silamiķelis, Kristaps Korņejevs, Daniella Borisova, Kaspars Megnis, Līga Eliņa, Vita Rovite, Annija Vaska, Kristaps Klavins, Helgi B. Schiöth, Janis Klovins

**Affiliations:** 1 Translational Omics Group, Latvian Biomedical Research and Study Centre, Riga, Latvia; 2 Faculty of Medicine and Life Sciences, University of Latvia, Riga, Latvia; 3 Institute of Biomaterials and Bioengineering, Faculty of Natural Sciences and Technology, Riga Technical University, Riga, Latvia; 4 Department of Surgical Sciences, Functional Pharmacology and Neuroscience, Uppsala University, Uppsala, Sweden

**Keywords:** Health sciences

## Abstract

Post-acute sequelae of COVID-19 (PASC) disproportionately affect hospitalized patients and require improved molecular characterization to inform patient management. Here, we performed a prospective longitudinal multi-omics study of hospitalized COVID-19 patients, analyzing whole blood transcriptomics, targeted urine metabolomics, kidney injury biomarkers, and electronic health record-based outcome stratification across acute illness, one-month, and three-month recovery time points. Interconnected immunothrombosis-related pathways dominated the acute phase, while most immune and metabolomic pathways partially normalize. However, patients who developed long COVID exhibited a distinct blood transcriptional signature at three months consistent with an endothelial-associated activation profile, including platelet reactivity, complement dysregulation, and low-grade vascular inflammation, distinguishing them from fully recovered individuals. This multi-omics approach identifies clinically measurable biomarkers associated with longitudinal molecular trajectories and supports post-acute risk stratification.

## Highlights

• Severe COVID-19 induces immunothrombosis-associated molecular programs
• Acute COVID-19 is associated with mitochondrial metabolic dysregulation
• Urine profiling indicates gradual renal recovery after hospitalization
• Long COVID patients retain endothelial-associated activation signatures

## Graphical abstract

**Figure:**
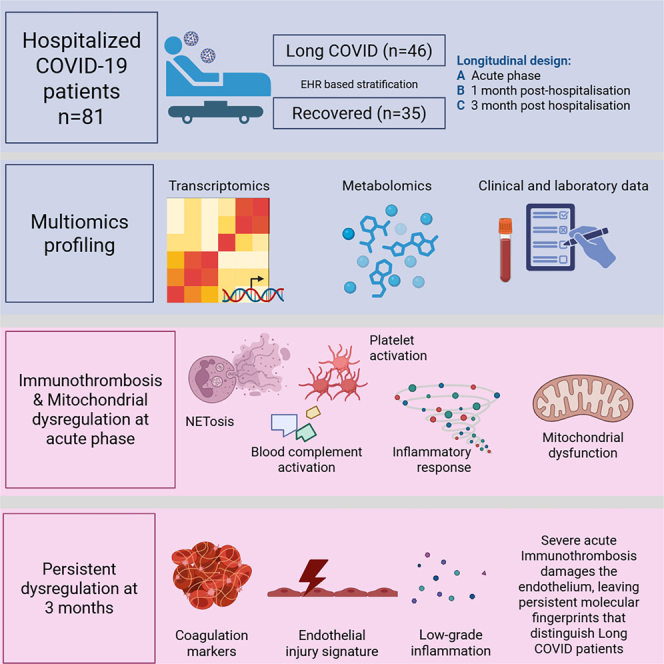

## Introduction

Due to the high transmissibility of SARS-CoV-2, COVID-19 rapidly progressed into a global health crisis of unprecedented scale in recent decades. Although, the widespread vaccination and ongoing viral evolution have since mitigated its severity and mortality, COVID-19 remains lethal for many. Sustained efforts to consolidate translational knowledge are therefore critical, particularly in the context of post-acute sequelae, where systems-level omics approaches can clarify disease mechanisms and guide more precise interventions. Post-acute sequelae of COVID (PASC)-19 (PASC), post-COVID-19 condition or long COVID is defined by persistence or emergence of new symptoms three months following the first SARS-CoV-2 infection, with these symptoms lasting for a minimum of two months without an alternative explanation.^1^

According to estimates, PASC affects approximately 10%–20% of individuals who tested positive for COVID-19, but data are still limited due to novel and heterogenous nature of the condition.^2^^,^^3^^,^^4^^,^^5^^,^^6^ USA study analyzing electronic health registry (EHR) data from two large cohorts, INSIGHT (35,275 COVID+ vs*.* 326,126 controls) and OneFlorida+ (22,341 COVID+ vs*.* 177,010 controls), found that within 30–180 days post infection, the most common PASC complications included pulmonary fibrosis, dyspnea, pulmonary embolism, chest pain, abnormal heartbeat, dementia, hair loss, pressure ulcers, malaise, and fatigue.^5^ Notably, PASC is significantly more prevalent in hospitalized patients, but it can also affect individuals who initially experienced only mild symptoms. Observational study of 1.2 million individuals in 22 countries that specifically stratified by hospitalization status, estimated that PASC incidence in ICU-admitted patients is 43.1%, hospitalized patients 27.5% and 5.7% in non-hospitalized COVID-19 patients.^2^ They also calculated that hospitalized patients tend to have PASC symptoms 9 months on average, but non-hospitalized 4 months.^2^ Most common symptoms in hospitalized patients are fatigue (53%–64%), dyspnea (26%–43%), sleep disturbances (24%–26%), joint/muscle pain (19%–27%), anxiety/depression (23%), chest pain (11%–22%).^3^^,^^4^^,^^6^ Moreover, acute kidney injury (AKI) emerges as one of the most prognostically important complications among hospitalized COVID-19 patients, affecting roughly one-third of cases and strongly predicting adverse post-acute outcomes.^7^ Pathological studies describe a consistent triad of acute tubular injury, collapsing glomerulopathy, and thrombotic microangiopathy.^7^^,^^8^

Regarding pathological mechanisms of long COVID, previous research indicates that multi-system complications in PASC arise from a complex interplay of mechanisms, including blood clotting abnormalities and endothelial damage, chronic immune dysregulation such as autoimmunity and immune cell exhaustion, and viral persistence or reactivation of latent viruses.^9^^,^^10^ Notably, SARS-CoV-2 infection triggered persistent, fibrinolysis-resistant amyloid fibrin microclots have been detected in PASC patients and these microclots are capable of seriously obstructing capillary blood flow.^9^^,^^11^ However, key questions remain about the causality and the hierarchy of these mechanisms.

The evidence supports risk-stratified post-COVID care, with hospitalized patients requiring more intensive and longer-duration follow-up protocols. Our previous metabolomics work revealed early disturbances in tryptophan-kynurenine metabolism during severe COVID-19, highlighting systemic metabolic reprogramming as a potential contributor to disease trajectory.^12^ Building on this, we implemented a longitudinal multi-omics approach integrating blood transcriptomics, urine metabolomics, and kidney injury biomarkers to track molecular trajectories from acute hospitalization through early and late recovery. This design enables distinction between transient responses to severe infection and persistent molecular alterations associated with post-acute sequelae. We show that the acute phase is marked by a profound immunothrombotic response, mitochondrial dysfunction with a shift toward glycolysis, and clear signs of tubular kidney stress. Although many of these processes begin to normalize within three months, patients who go on to develop long COVID display a distinct transcriptional profile characterized by sustained endothelial-associated and platelet activation signatures, complement imbalance, and low-grade inflammation.

## Results

### Cohort overview and EHR-based outcome stratification

This study employs a prospective design with longitudinal sampling to examine whole blood transcriptome, urine metabolite and urine kidney damage marker profiles in severe COVID-19 patients across three time points: (A) 1st day of hospital admission, (B) one month (38.46 ± 12.89 days) post-hospitalization, and (C) three month recovery (96.29 ± 13.81 days post-hospitalization). We performed whole blood transcriptomics from 81 hospitalized COVID-19 patient (Table 1). Five patients (6.2%) required intensive care unit (ICU) admission during hospitalization; all ICU admitted patients received supplemental oxygen therapy, but none required invasive or non-invasive mechanical ventilation. It should be noted that 62 patients had blood collected at all three time points (19 patients have transcriptome data from two time points (acute phase [time point A] and recovery phase [time point B or C]), 224 blood transcriptomes in total. Additionally for 61 of these patients with available urine sample (183 samples across all time points) targeted LC-MS urine metabolomics (46 analytes) and LUMINEX platform based kidney damage biomarker detection was performed. More phenotypical information of 61 patient sub-cohort can be found in Table S1.

**Table 1:** Cohort demographics and clinical characteristics of 81 hospitalized, severe COVID-19 patient

Variable, mean (SD) or n (%)	Recovered from COVID-19 (N = 35)	Long COVID (N = 46)
Male/Female (n, %)	15 (42.86%)/20 (57.14%)	20(43.48%)/26 (56.52%)
Age, average ±SD (years ±SD)	55.03 ± 15.59	59.22 ± 12.71
BMI, average (kg/m2 ± SD)	27.81 ± 6.40	31.14 ± 6.32
Hospitalization days (days ±SD)	9.45 ± 4.77	11.47 ± 5.28
Smoker/non-smoker (n, %)	3 (8.57%)/32 (91.43%)	5(10.87%)/41 (89.13%)

***Comorbidities before hospitalization***^a^		

**Number of patients with comorbidities (yes/no)**	18 (51.43%)/17 (48.57%)	23 (56.10%)/18 (43.90%)
Hypertension (n, %)	10 (28.57%)	18 (39.13%)
Type 2 Diabetes Mellitus (n, %)	2 (5.71%)	5 (10.87%)
Other cardiovascular disease (n, %)	4 (11.43%)	15 (32.61%)
Oncological (n, %) Other	2 (5.71%) 4 (11.43%)	5 (10.87%) 10 (27.03%)

**New comorbidities in post-acute hospitalized COVID-19 in EHR for Long COVID group (N = 46)**^b^	

**Subgroup (n, %)**	**ICD-10 codes**
Respiratory (22, 47.83%)	J12.8, J18, J18.9, J43, J44, J44.9, J45.8, J84, J98, J98.4, R07.4
General or Musculoskeletal (15, 32.61%)	M13, M47, M47.2, M47.8, M50.1, M51.1, M54.1, M54.6, M75.4, M77.0, U09, U09.9
Nervous (12, 26.09%)	G43, G44, G45, G59.0, R00, R51
Circulatory (12, 26.09%)	I25, I67.9, I69, I69.3, I74.3
Genitourinary (10, 21.74%)	K80, N18.3, N30, N39
Digestive (7, 15.22%))	K31.9, K56.7, K74
Endocrine (3, 6.38%))	E11

**Clinical measurements for hospitalized COVID-19 cohort (N = 81)**^c^			

**Average (SD)**	**Acute COVID-19**	**Recovery phase (30 days)**	**Recovery phase (3–6 months)**
Leukocytes (μL)	5.74 (3.60)	6.81 (4.93)	6.25 (3.78)
Hemoglobin (g/dL)	13.49 (1.35)	13.68 (1.28)	13.99 (1.35)
Hematocrit (%)	43.44 (24.85)	41.42 (3.52)	42.06 (3.77)
Platelets (μL)	188.79 (67.15)	269.68 (58.53)	245.19 (54.28)
Neutrophils (μL)	2.45 (2.28)	3.27 (1.05)	3.06 (1.23)
Lymphocytes (μL)	0.90 (2.67)	2.51 (4.22)	2.38 (3.00)
Monocytes (μL)	0.24 (0.22)	1.37 (5.02)	0.50 (0.18)
Eosinophils (μL)	0.21 (1.31)	0.18 (0.13)	0.18 (0.12)
ALT (U/l)	33.96 (25.35)	33.74 (22.23)	29.51 (17.01)
AST (U/l)	43.51 (33.91)	27.03 (12.04)	25.84 (9.99)
GGT (U/l)	63.38 (60.38)	43.53 (38.99)	35.70 (29.95)
Bilirubin (μmol/L)	9.04 (8.05)	12.33 (5.50)	12.55 (11.77)
LDH (U/L)	309.09 (150.80)	217.46 (41.57)	202.95 (36.67)
Creatinine ((μmol/L)	74.86 (22.31)	69.72 (16.56)	72.67 (21.45)
CRP (mg/L)	56.23 (64.79)	3.52 (5.12)	2.37 (3.84)
D-dimer (mg/mL)	22.00 (108.56)	1.51 (5.32)	0.61 (1.01)

EHR, Electronic health registry; SD, standard deviation; BMI, body mass index; ALT, alanine aminotransferase; AST, aspartate aminotransferase, GGT, gamma-glutamyl transferase; LDH, lactate dehydrogenase; CRP, C-reactive protein.

a Comorbidities for population controls were self-reported.

b Each patient can be in more than one subgroup.

c Clinical measurements in Acute COVID-19 phase were performed at an in-house hospital clinical laboratory, but Recovery phase measurements in a certified clinical laboratory outside hospital.

**Figure 1:**
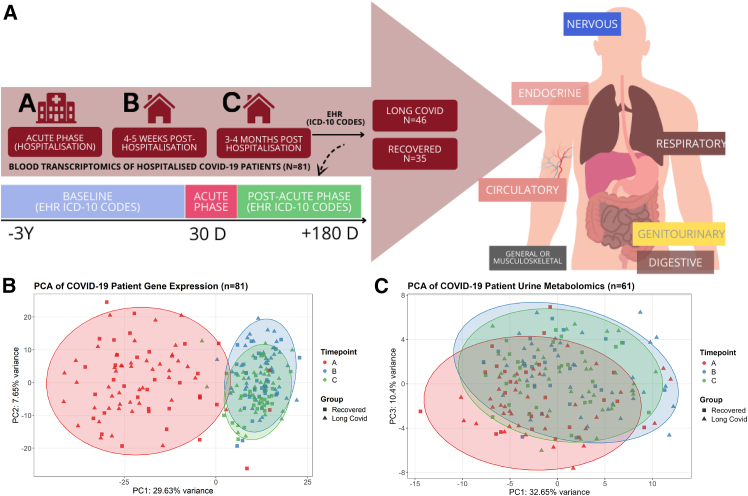
Omics data of hospitalized COVID-19 patients show that acute phase (time point A) samples separate distinctly from recovery phases (time points B and C), with extensive overlap between long COVID and recovered groups (A) Study design. Longitudinal samples were collected at hospital admission (time point A), one month (time point B), and three months (time point C) post-hospitalization. Electronic health registry (EHR) data were obtained for all patients (*n* = 81), who were classified as recovered (N = 35) or long COVID (N = 46), based on the presence of post-acute sequelae of COVID-19 (PASC) diagnoses within 12 months following the acute phase, according to criteria adapted from Zang et al.^5^ Subgroups were assigned based on post-acute EHR ICD-10 codes (see Table 1). (B) Time point (A-red, B-blue, and C-green) clustering demonstrates that acute/recovery phases dominates whole blood transcriptomic variance regardless of PASC outcome (square- recovered, triangle- long COVID). Unsupervised principal component analysis (PCA) (with 95% confidence ellipses) of blood transcriptome profiles from COVID-19 patients (N = 81) using the top 1000 most variable genes across three time points. Variance explained by PC1: 29.63%, PC2: 7.65%, and PC3: 4.72%. (C) Acute phase (time point A) samples separate distinctly from recovery phases (time points B and C) in targeted urine metabolome unsupervised PCA plot (with 95% confidence ellipses) of 46 targeted urine metabolites from COVID-19 patients (N = 61) across three time points (PC1: 32.65%, PC2: 12.52%, PC3: 10.40%). EHR-electronic health registry, PCA-principal-component analysis.

The enrolled patients were stratified, based on EHR data in two phenotypes (long COVID, recovered), by the following criteria: (1) long COVID patients had a new PASC diagnosis in 12 months following COVID-19 acute phase (1-month acute phase, 12 month period as a post-acute period), that the patient did not had in baseline period (3 years before acute COVID-19 phase); (2) recovered patients did not have a new PASC diagnosis in 12 months following acute phase (Figure 1). For this stratification we used PASC diagnosis list from the recently published work of Zang et al.,^5^ that included 137 ICD-10 codes and also stratified patients by affected organ system complications. Thus, we classified 81 patients in two groups: long COVID (N = (46) and recovered (N = 35). As shown in Table 1, patients who later developed long COVID were on average older, had higher BMI, and experienced slightly longer hospitalization duration, indicating modest differences in baseline severity characteristics between groups. Post-acute symptoms for long COVID group were classified as respiratory (*n* = 22, 48%), general/musculoskeletal (N = 15, 33%), nervous system (N = 12, 26%), circulatory (N = 12, 26%), genitourinary (*n* = 10, 22%), digestive (N = 7, 15%), endocrine (N = 3, 7%). Many subjects have multiple symptom categories, so percentages sum to >100% (Table 1; Figure 1A).

**Figure 2:**
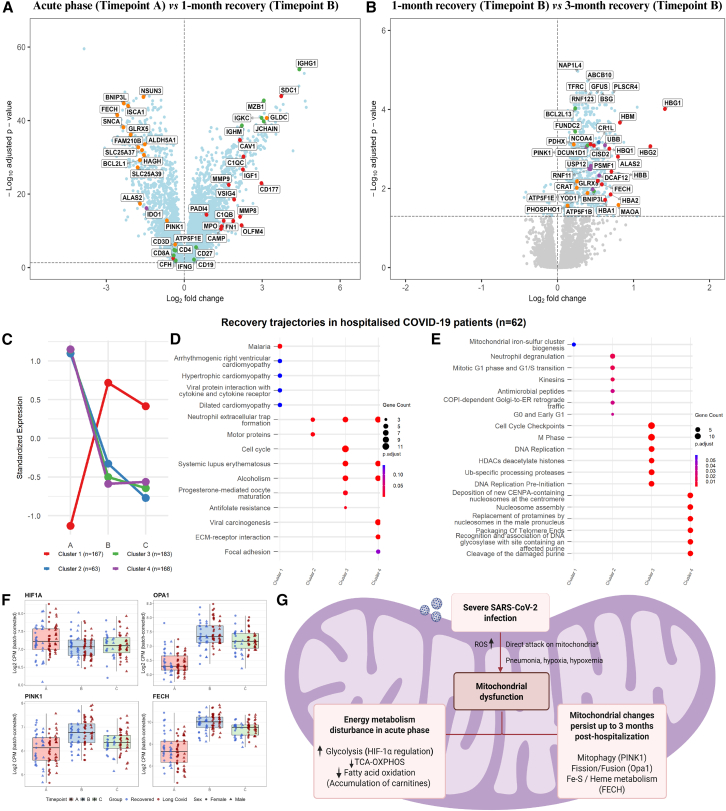
Longitudinal molecular changes in blood cells during recovery from acute COVID-19 show significant transcriptomic remodeling with pronounced dysregulation of energy metabolism genes and immune activation (A) Volcano plot of blood transcriptome differences between acute COVID-19 (time point A) and 1-month recovery (time point B). The *x* axis represents log_2_ fold change (A vs. B), and *y* axis shows -log_10_(FDR adjusted *p* value), light blue dots—statistically significant (FDR <0.05) differentially expressed genes (DEGs), gray—not significant, some genes are highlighted: red—immunothrombosis genes, orange—mitochondrial function genes, green—T and B cell response genes, purple—IDO1 gene. (B) Volcano plot of blood transcriptome differences between 1-month (time point B) and 3-month recovery (time point C). The *x* axis represents log_2_ fold change (B vs. C), and *y* axis shows -log_10_(FDR adjusted *p* value), light blue dots—statistically significant (FDR <0.05) genes, gray—not significant, some genes are highlighted, red-heme metabolism and mitochondrial iron metabolism genes, blue-immunity and inflammation genes, orange-energy metabolism genes, green—mitophagy genes, purple-proteostasis and ubiquitination genes. Differential expression analysis was performed using *limma* linear modeling with empirical Bayes moderation; *p* values were adjusted using the Benjamini-Hochberg method (FDR <0.05). (C) Temporal *Mfuzz* clustering (k = 4) of transcriptomic trajectories in hospitalized COVID-19 patients (*n* = 62) who had transcriptome data from all 3 time points. (D and E) (D) Dotplots showing KEGG and (E) reactome pathway enrichment analysis of cluster-specific genes, with dot size and color representing gene count and significance, respectively. Pathway enrichment analysis was performed using *clusterProfiler* with Benjamini-Hochberg FDR correction. (F) Differential expression of HIF1A, PINK1, OPA1, FECH genes across time points (A-hospital admission, B- 1-month post hospitalization, C- 3-month recovery). Boxplots display batch-corrected log_2_CPM expression values. Each box represents the interquartile range (IQR) with median line; whiskers extend to 1.5 × IQR. Individual data points correspond to samples, colored by clinical group (long COVID = red, recovered = blue) and shaped by sex (females = circles, males = triangles). Differential expression across time points was assessed using linear mixed-effects modeling implemented in limma with subject as a blocking factor; *p* values were adjusted using the Benjamini-Hochberg method (FDR <0.05). *n* = 62 patients. (G) Summary of SARS-CoV-2 caused mitochondria damage signals detected by whole blood transcriptomics, ∗SARS-CoV-2 direct attack on mitochondria has been showed by Zong et al.^13^ In hospitalized severe cases the infection causes severe pneumonia with hypoxemia and hypoxemia, the reaction to these circumstances are regulated by HIF-1A. Gene expression analysis show “rebound-like’’ trajectory for mitochondrial genes related to mitophagy (e.g., PINK1), mitochondrial membrane dynamics—fision/fusion (e.g., OPA1), Fe-S and Heme metabolism (e.g., FECH). Created in BioRender https://BioRender.com/po3eo9g. HIF1A-hypoxia-inducible factor 1-alpha, PINK1-PTEN-induced kinase 1, OPA1-optic atrophy 1, FECH-ferrochelatase.

**Figure 3:**
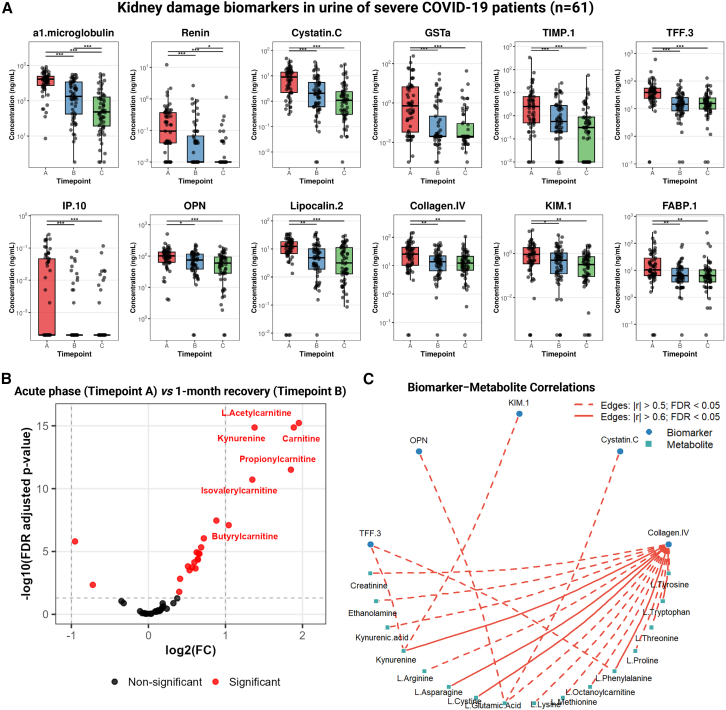
Urine omics (targeted metabolomics, multiplex immunoassay of kidney damage biomarkers) of hospitalized COVID-19 patients (N = 61) indicate renal recovery (A) Kidney damage biomarkers in urine during recovery. Boxplots showing longitudinal changes in significantly altered kidney injury biomarkers across time points A, B, and C (*x* axis), *y* axis shows biomarker concentrations in ng/mL (log_10_ scale applied), asterisks denote significance (∗FDR <0.05; ∗∗FDR <0.01; ∗∗∗FDR <0.001). Statistical significance was assessed using linear mixed-effects modeling implemented in *limma* with subject as a blocking factor; *p* values were adjusted using the Benjamini-Hochberg method. GSTa-glutathione S-transferase alpha, TIMP1-tissue inhibitor of metalloproteinases-1, TFF3-trefoil factor 3, IP10-interferon-gamma-induced protein 10, OPN-osteopontin, KIM1-kidney injury molecule-1, FABP1-fatty acid-binding protein 1. (B) Volcano plot of urine metabolite changes between acute phase (time point A) and 1-month recovery (time point B). Targeted LC-MS metabolomics analysis (46 metabolites), red points indicate metabolites with significant changes (FDR-adjusted *p* value <0.05). Differential expression analysis was performed using limma linear modeling with empirical Bayes moderation; *p* values were adjusted using the Benjamini-Hochberg method (FDR <0.05). (C) Biomarker-metabolite correlations. Circular network visualization of significant correlations between urinary kidney injury biomarkers (red circles) and serum/urine metabolites (blue squares). Edges represent Spearman correlations with absolute correlation coefficient |r| > 0.5 or |r| > 0.6 for the statistically significant associations (FDR <0.05). TFF3-trefoil factor 3, OPN-osteopontin, KIM1-kidney injury molecule-1.

**Figure 4:**
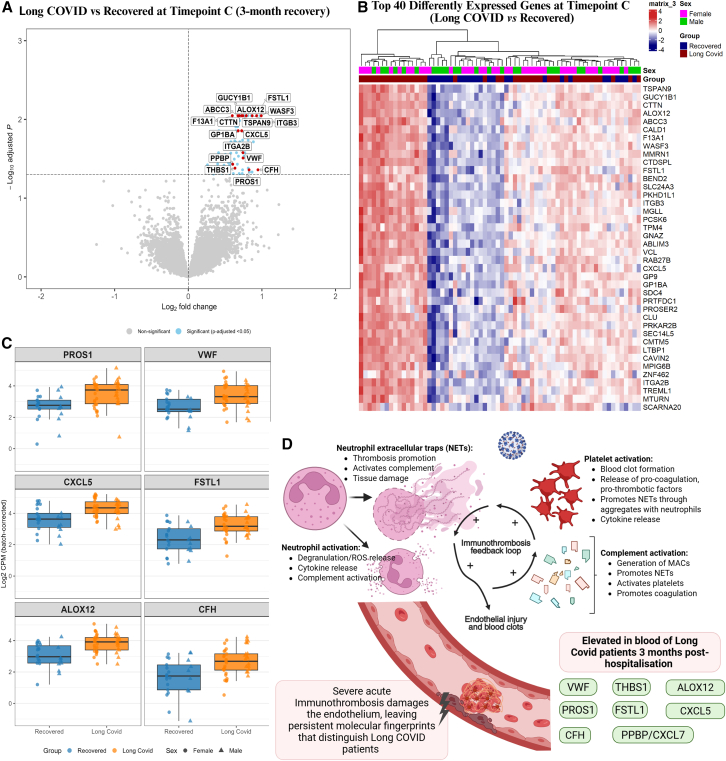
Differences in blood transcriptomes between patients with PASC/long COVID and hospitalized patients who fully recover indicate endothelial-associated and platelet activation signatures, including increased Von Willebrand factor (VWF) and PROS1 (protein S) (A) Long COVID vs*.* recovered transcriptomic differences at time point C. Volcano plot showing differential gene expression analysis between long COVID (N = 46) and recovered (N = 35) patients at 3-month recovery. The *x* axis represents log_2_ fold change (long COVID vs. recovered), *y* axis shows -log_10_(adjusted *p* value) with Benjamini-Hochberg FDR correction. Differential expression analysis was performed using limma linear modeling with empirical Bayes moderation. Significant genes (FDR-adjusted *p* value <0.05) are shown in light blue; highlighted genes represent selected molecular functions/pathways. (B) Heatmap of TOP 40 statistically differently expressed genes between long COVID and recovered groups 3 months post-hospitalization (time point C). (C) Boxplots of PROS1, VWF, CXCL5, FSTL1, ALOX12, and CFH at time point C (3 months post hospitalization). Boxes represent the interquartile range (IQR) with the median indicated by the central line; whiskers extend to 1.5×IQR. Individual points represent patient samples and are colored by clinical group (long COVID = red, recovered = blue) and shaped by sex (circles = female, triangles = male). Differential expression between groups was assessed using limma linear modeling with empirical Bayes moderation; *p* values were adjusted using the Benjamini-Hochberg method (FDR <0.05). Long COVID (N = 46), recovered (N = 35). (D) Schematic representation of immunothrombosis-related mechanisms, including interactions between NETosis, platelet activation, and complement activation, and their association with endothelial injury signals detected up to three months post-hospitalization in long COVID patients. Created in BioRender: https://BioRender.com/p4ytvcq. PASC - post-acute sequelae of COVID-19, VWF-Von Willebrand factor, PROS1-protein S, CXCL5-C-X-C motif chemokine 5, FSTL1-follistatin-related protein 1, ALOX12-arachidonate 12-lipoxygenase, CFH-complement factor H.

The physiological changes during recovery from severe COVID-19 create large-scale molecular shifts regardless of whether patients develop PASC/long COVID. This is clearly evident in the PCA plots of both transcriptomic (Figure 1B) and metabolomic (Figure 1C) datasets, where the largest source of variance is driven by the time point rather than the clinical outcome group. In both omics layers, samples from the acute phase (A) form distinct clusters that shift markedly toward the recovery phases (B and C), indicating common longitudinal remodeling trajectories shared across the cohort and supporting a recovery-first analytical approach of all hospitalized COVID-19 patients (Figure 2; Figure 3), before looking for differences between long COVID and recovered groups (Figure 4).

### Recovery from severe COVID-19 takes at least 3 months and can be detected through specific changes in the blood transcriptome and urine metabolome

During the acute-to-recovery transition (time point A vs*.* B), 10633 genes showed significant differential expression (FDR < 0.05, Figure 2A; Table S2A), the late recovery phase (time point B vs. C) demonstrated significant dysregulation of 678 genes (Figure 2B; Table S2B). Targeted urine metabolomics analysis (46 quantified metabolites) identified 23 statistically significant metabolites (FDR < 0.05) in acute-to-recovery transition (time point A vs*.* B, Figure 3B; Table S2C), but none were significant in the late recovery phase (time point B vs. C). Multiple urine kidney damage biomarkers demonstrated significant (FDR < 0.05) longitudinal variation (Figure 3A; Table S2D). Fuzzy clustering analysis using optimized parameters (k = 4, m = 3.773) was performed for hospitalized COVID-19 patients who had all three time point (A, B, and C) transcriptome data and it identified distinct temporal gene expression trajectories that provide additional insights into molecular recovery patterns (Figure 2C; Table S2E), KEGG and reactome pathway analysis results of each cluster are also displayed in Figures 2C–2E, (Table S2F and S2G).

#### Severe SARS-CoV-2 infection causes mitochondrial dysfunction with Warburg-like metabolism in acute phase and detectable mitophagy signals in later recovery

##### Hypoxemia during acute SARS-CoV-2 infection in hospitalized patients triggers HIF-1α activation, shifting cells toward glycolysis and away from OXPHOS

During the acute-to-recovery transition (time point A vs*.* B), whole-blood transcriptomics revealed extensive downregulation of mitochondrial oxidative phosphorylation (OXPHOS) genes and upregulation of glycolytic and hypoxia-responsive pathways (FDR<0.05; Figure 2A). Key transcriptional regulators of cellular hypoxia, HIF1A and EPAS1 (HIF-2α), together with their canonical targets BNIP3L, PFKFB3, ARNT, EGLN1, and VHL, were significantly upregulated in acute phase, consistent with hypoxemia-driven HIF-1α activation. Several glycolysis genes, including GAPDH, ENO1, HK1, PGK1, PFKP, and HK2, were strongly upregulated, whereas multiple TCA and OXPHOS components were repressed (ATP5F1B, ATP5F1E, NDUFA4, NDUFB6, COX7A1, and COX6A2) suggesting a metabolic rerouting toward anaerobic ATP production reminiscent of the Warburg effect. The PDK1 and LDHA expression pattern supports inhibition of pyruvate oxidation and lactate accumulation. Concordantly, targeted urine metabolomics (Figure 3B; Table S2C) identified elevated acylcarnitines (FDR<0.05) in acute phase, L-acetylcarnitine (log_2_FC = 1.8), carnitine, propionylcarnitine, isovalerylcarnitine, butyrylcarnitine, and L-octanoylcarnitine—indicating reduced mitochondrial β-oxidation and disruption of the carnitine shuttle. None of the quantified metabolites were significantly different between one- and three-month recovery (B vs*.* C), implying partial metabolic stabilization.

##### Mitophagy and mitochondrial fission/fusion regulators show “rebound-like’’ expression trajectory

In the late-recovery interval (B vs*.* C), 678 genes remained significantly dysregulated (FDR < 0.05; Figure 2B), highlighting persistent mitochondrial remodeling. Notably, mitophagy regulators PINK1 (log_2_FC = 0.39, Figure 2F), BCL2L13, and FUNDC2 exhibited significant upregulation in time point B and then downregulation in time point C, together with mitochondrial fusion gene OPA1 (Figure 2F), this expression behavior is reflecting enhanced mitochondrial turnover and repair. Energy metabolism genes such as ATP5F1E, CRAT, and PDHX showed similar rebound expression patterns, consistent with reactivation of oxidative metabolism. Also, a coordinated transcriptional upregulation of heme biosynthesis and mitochondrial iron-sulfur cluster genes were significantly different, including FECH and ALAS2 genes (Figure 2F). Upregulation of FECH, the terminal rate-limiting enzyme inserting ferrous iron into protoporphyrin IX, together with ALAS2, the erythroid-specific initiator of heme synthesis, indicates renewed mitochondrial heme production. This transcriptional activation was paralleled by elevated hemoglobin subunit genes (HBG1, HBG2, HBM; log_2_FC = 0.8–1.4; FDR < 0.01), indicating recovery of oxygen-carrying capacity and redox homeostasis.

Mfuzz clustering (k = 4, m = 3.77) identified cluster 1 as a trajectory characterized by suppressed expression in the acute phase (time point A) followed by a progressive rebound through one- and three-month recovery (A→C) (Figure 2C). This cluster included key regulators of mitochondrial quality control and energy metabolism, such as PINK1, BCL2L13, and FUNDC2, which mediate mitophagy; the mitochondrial fusion factor OPA1; metabolic enzymes ATP5F1E, CRAT, and PDHX; and genes involved in heme biogenesis (FECH) all showing synchronized recovery of expression (Figures 2D–2F; Table S2E). Collectively, cluster 1 illustrates a “rebound-like” molecular recovery program coupling mitophagy, mitochondrial fusion, and metabolic reactivation during convalescence. These transcriptional dynamics mirror the conceptual model in Figure 2G, in which acute SARS-CoV-2-induced mitochondrial damage is followed by gradual mitochondrial renewal and re-establishment of redox balance up to at least three months post-hospitalization.

#### Interconnected immunothrombotic pathways dominate the acute phase of severe COVID-19

##### Blood complement system activation in acute COVID-19 contributes to coagulation

The complement system, a key effector of innate immunity that facilitates opsonization, inflammation, and downstream coagulation signaling, showed extensive transcriptional activation during the acute phase (time point A). Genes encoding the classical pathway initiators C1QA (log_2_FC = 0.93), C1QB (1.52), and C1QC (2.28) were among the most significantly upregulated (FDR <0.001), together indicating C1-complex activation. Downstream, the C5AR1 receptor (log_2_FC = 0.35) was also elevated, consistent with enhanced C5a-driven leukocyte and platelet recruitment. The complement regulator CFH was transiently suppressed during the acute phase (−0.42, FDR <0.05) and rosed during recovery. CFH acts by binding C3b and preventing uncontrolled complement amplification on host surfaces; its downregulation during the acute phase likely permits unrestrained complement turnover.

##### Neutrophil activation and excessive NETosis mark the acute inflammatory response

Neutrophils were among the most transcriptionally active immune cell populations during the acute-to-recovery transition (A vs*.* B), showing strong induction of NETosis and degranulation programs (Figure 2A). Neutrophil extracellular traps (NETs) are networks of extracellular fibers that bind and neutralize pathogens, they are primarily composed of DNA, proteases, and RNAses from neutrophils. While NETs participate in pathogen capture, excessive NET formation drives persistent immunothrombotic complications. Key NETosis regulators were significantly upregulated during the acute-to-recovery transition (A vs. B, FDR <0.001), including PADI4, MPO, and CAMP accompanied by strong induction of neutrophil degranulation markers CD177 (logFC = 2.97), OLFM4 (2.21), and MMP8 (2.15). These expression changes reflect widespread neutrophil activation and cytotoxic mediator release in circulation. Importantly, fuzzy c-means clustering (mfuzz) analysis (k = 4, m = 3.77) identified “neutrophil extracellular trap formation” as the only KEGG pathway enriched across three of four temporal clusters (Figure 2C), highlighting NET formation as a core longitudinal signal spanning distinct recovery trajectories.

##### Sustained platelet activation reinforces prothrombotic signaling

Platelets, which normally ensure hemostasis, showed transcriptional hallmarks of heightened aggregation and vascular interaction potential (Figures 2A and 2B). Key platelet surface receptors—ITGA2B (αIIb), ITGB3 (β3), and GP1BA—were significantly increased (FDR <0.01) in acute phase. Several downstream effectors (THBS1, PPBP, and F13A1) remained upregulated during the one-to three-month interval (B vs*.* C, FDR <0.05), suggesting incomplete normalization of platelet reactivity after hospital discharge. This persistent expression pattern, together with complement and NETosis signatures, defines a feedforward immunothrombotic loop, in which complement activation recruits and primes neutrophils, neutrophils release NETs that trap and activate platelets, and platelets amplify coagulation and complement signaling. These interconnected pathways together shape the molecular landscape of acute severe COVID-19 and provide a mechanistic basis for the increased vascular complication risk (Figure 4D).

#### Dysregulated tryptophan-kynurenine metabolism links immune activation to energy imbalance

The rate-limiting enzyme indoleamine 2,3-dioxygenase 1 (IDO1) was significantly downregulated in the blood cells during acute phase (log_2_FC = −1.45, FDR <0.01, Figure 2A) opposed to 1-month recovery phase, yet its downstream metabolite kynurenine was elevated in urine (log_2_FC = 1.38, FDR <0.01) during the same interval (A vs*.* B, Figure 3B). The dissociation between reduced blood IDO1 transcription and elevated urinary kynurenine likely reflects spatial compartmentalization of interferon-driven tryptophan metabolism. During acute SARS-CoV-2 infection, IDO1 is strongly induced by type I and II interferons at the primary site of infection, the respiratory system, where it converts tryptophan to kynurenine. Ongoing interferon-driven IDO1 activity in infected respiratory tissues may therefore have generated substantial kynurenine that subsequently accumulated in circulation and was excreted in urine. Thus, reduced IDO1 mRNA levels in whole blood at admission do not indicate absence of pathway activation, but instead reflect tissue-specific regulation of IDO1 expression. The tryptophan-kynurenine pathway provides the *de novo* source of NAD^+^ synthesis from tryptophan. Systemic activation of the tryptophan-kynurenine pathway connects immune signaling with redox and energetic stress.

#### Urinary kidney injury biomarkers and metabolite signatures show renal recovery after severe COVID-19

Longitudinal analysis of 13 kidney injury biomarkers using multiplex immunoassay revealed eleven significantly changed biomarkers across time points (FDR <0.05) (Figures 3A; Table S2D). Classical tubular stress indicators, like α1-microglobulin, cystatin C, lipocalin-2, and kidney injury molecule-1 (KIM-1) showed strong acute-phase elevation (log_2_FC 1.8–2.6, A vs*.* B FDR <0.001) followed by progressive decline, consistent with tubular recovery. Inflammatory and fibrotic mediators such as osteopontin (OPN), interferon-gamma-induced protein 10 (IP-10), tissue inhibitor of metalloproteinases-1(TIMP-1), and glutathione S-transferase alpha (GST-α) also peaked during the acute phase (FDR <0.001). By contrast, renin and α1-microglobulin remained significantly reduced between one- and three-month recovery (B vs*.* C, FDR <0.05), suggesting delayed normalization of renal hemodynamic and reabsorptive function. Other markers, including trefoil factor 3 (TFF-3) and collagen IV, were significantly elevated in the acute phase (A vs. B, FDR <0.01) but stabilized thereafter.

Targeted LC-MS metabolomics of 46 urinary metabolites (Figure 3B) demonstrated parallel metabolic remodeling. In the acute-to-recovery transition (A vs*.* B), 23 metabolites were significantly altered (FDR <0.05). Acylcarnitines, including L-acetylcarnitine (log_2_FC = 1.8), carnitine, propionyl-, isovaleryl-, and butyrylcarnitine, were significantly elevated during the acute phase, reflecting impaired mitochondrial β-oxidation. Amino acids such as L-serine, L-asparagine, and L-glutamine increased during recovery, indicating restoration of oxidative and nitrogen metabolism. No significant metabolite changes were detected between one- and three-month recovery (B vs*.* C), consistent with metabolic stabilization.

Integrative correlation analysis (Figure 3C) identified multiple significant biomarker-metabolite pairs (FDR <0.05). Collagen IV (FDR <0.01), essential for basement membrane integrity and filtration barriers, emerged as a central hub in the kidney-metabolite interaction network, demonstrating 40 significant correlations, the strongest correlation was with kynurenine (r = 0.72, FDR <0.05), L-phenylalanine (0.67), L-tryptophan (0.64), and L-tyrosine (0.58). These observations suggest that kidney basement membrane integrity is connected to systemic immunometabolism dysfunction.

### Persistent endothelial-associated and immunothrombotic activation signatures define hospitalized COVID-19 patients with post-acute sequelae (long COVID)

The three-month recovery transcriptomes (time point C) revealed significant divergence between long COVID and recovered patients (FDR <0.05; Figure 4A; Table S3A). No differences reached statistical significance after FDR correction at earlier time points or in urine metabolomics and kidney biomarker profiles, but several urine metabolites exhibited nominal associations (raw *p* < 0.05; Table S3B). We also performed sparse canonical correlation analysis (SCCA) to identify possible transcriptome or metabolite markers associated with complications, but this analysis did not yield statistically significant results. The heatmap of the top 40 differently expressed genes (DEGs) (Figure 4B) shows partial overlap of long COVID and Recovered patients, reflecting the heterogeneity of post-acute outcomes observed clinically (Table 1).

#### Sustained endothelial-associated and platelet activation signatures mark long COVID blood transcriptomes

Genes associated with endothelial and platelet activation remained significantly elevated in long COVID patients at three-month recovery (FDR <0.05; Figure 4A). Von Willebrand factor (VWF, log_2_FC = 0.744) and the natural anticoagulant protein S (PROS1, log_2_FC = 0.(652) were both upregulated (Figure 4C), consistent with persistent vascular perturbation and altered pro- and anticoagulant balance detectable in blood. As VWF transcripts in whole blood may originate from megakaryocytes and platelets, these changes likely reflect altered platelet abundance, turnover, or activation state rather than direct endothelial gene expression. Platelet receptor subunits ITGA2B (αIIb), ITGB3 (β3), and GP1BA, together with the clot-stabilizing factor F13A1, were significantly increased, consistent with persistent platelet priming and hypercoagulability (Figure 4A). Elevated THBS1 and PPBP further indicated platelet activation and enhanced platelet-leukocyte communication (Figure 4A). Collectively, these findings identify a sustained prothrombotic transcriptional state with endothelial-associated features as a key molecular hallmark distinguishing long COVID cases from fully recovered patients. These findings are summarized in the pathological model presented in Figure 4D.

#### Persistent inflammatory signaling and complement dysregulation in long COVID

Long COVID transcriptomes demonstrated upregulation of genes implicated in neutrophil recruitment and lipid-mediated inflammation (FDR <0.05). CXCL5 (log_2_FC = 0.725), a potent neutrophil chemoattractant and angiogenic chemokine, remained significantly elevated, indicating ongoing low-grade inflammatory signaling (Figure 4C). ALOX12 also was upregulated in long COVID cases (log_2_FC = 0.787), it encodes 12-lipoxygenase that catalyzes the formation of 12-HETE—a pro-inflammatory lipid mediator (Figure 4C). Its upregulation reflects persistent platelet activation, neutrophil recruitment, endothelial dysfunction, and oxidative stress—key features of chronic vascular inflammation. The complement regulator CFH (log_2_FC = 0.946) showed compensatory elevation, consistent with attempts to restrain chronic complement activation observed during the acute phase (Figure 4C). Together, these transcriptional markers indicate chronic low-grade inflammation in long COVID patients.

#### Ongoing tissue remodeling and vascular repair programs in long COVID

Markers of cytoskeletal remodeling and fibrotic repair were among the most upregulated genes in Long COVID blood transcriptomes (FDR <0.05). WASF3 (log_2_FC = 0.991) and CTTN (0.694), regulators of actin polymerization and cellular motility, indicate enhanced cell-adhesion and migration programs (Figures 4A and 4B). FSTL1 (0.928), a secreted glycoprotein promoting myofibroblast differentiation and extracellular matrix deposition (Figure 4C), and THBS1 (0.634), an anti-angiogenic matricellular protein involved in TGF-β activation, both pointed to active tissue remodeling processes (Figure 4A). The co-upregulation of cytoskeletal and matrix-associated genes suggests ongoing vascular and interstitial repair responses three months after severe infection, potentially reflecting incomplete resolution of endothelial injury.

## Discussion

In this longitudinal multi-omics study of hospitalized COVID-19 patients, we identify immunothrombosis-related molecular programs as a central feature associated with post-acute complication risk. Our omics analyses show that severe SARS-CoV-2 infection induces a coordinated cascade of complement activation, neutrophil extracellular trap (NET) formation, platelet priming, mitochondrial dysfunction, and renal tubular stress. While many metabolic and inflammatory pathways partially normalize within three months, patients who develop long COVID retain a distinct blood transcriptional signature marked by persistent endothelial-associated and platelet activation signatures, complement regulation and low-grade inflammation. Based on these findings, we propose a mechanistic model in which acute immunothrombosis leaves a durable endothelial-associated molecular imprint detectable in patients with long COVID risk (Figure 4D). Importantly, several components of this signature, including VWF and PROS1, represent clinically measurable blood biomarkers, highlighting their potential utility for post-acute risk stratification and mechanistic disease monitoring.

Our finding suggests that immunothrombosis represents a convergence point between innate immunity and coagulation and it can lead to vascular injury in hospitalized patients with PASC. While causality cannot be directly inferred from observational blood-based omics data, the persistence of these signatures supports immunothrombosis as a key biological axis in post-acute COVID-19 pathology. The severity of immunothrombosis seems to be driven by complement-NET-platelet interactions form a self-reinforcing loop: complement fragments activate neutrophils, NETs provide a scaffold for platelet aggregation and coagulation, and activated platelets further amplify complement signaling. Moreover, our data show that three months after acute infection, individuals with PASC retained elevated expression of endothelial-associated activation markers (VWF and PROS1) and inflammatory mediators (ALOX12, CXCL5, and CFH); therefore, our findings indicate a persistent blood transcriptional signature consistent with endotheliopathy and thromboinflammation in long COVID. Thus, recognizing the pathological cause and appropriately monitoring hospitalized patients would enable risk-stratified intervention. These findings are supported by Bonaventure et. Al review published in Nature reviews.^14^ Interestingly, previous vascular function studies demonstrate persistently impaired flow-mediated dilation (FMD) for months after SARS-CoV-2 infection, with the greatest deficits observed in individuals who experienced severe or hospitalized disease.^15^^,^^16^ Taken together, it seems plausible that the ongoing microvascular leak, impaired glycocalyx regeneration, persistent complement activation, and platelet priming as plausible contributors to post-acute symptom clusters including fatigue, exertional intolerance, dyspnea, and cognitive dysfunction.^17^^,^^18^ Moreover, compensatory upregulation of complement regulators such as CFH aligns with recent multi-omics evidence of sustained alternative complement pathway activation in active long COVID.^19^

Our findings suggest that severe SARS-CoV-2 infection imposes a profound bioenergetic stress characterized by hypoxia-driven metabolic reprogramming and mitochondrial dysfunction. The observed transcriptome profile in blood cells is consistent with a systemic Warburg-like metabolic shift. This state has important inflammatory consequences: (1) hypoxia-driven glycolysis in endothelial cells promotes vasoconstriction and microthrombosis, (2) glycolysis sustains pro-inflammatory neutrophils and M1 macrophages, which rely on aerobic glycolysis to maintain effector function.^20^^,^^21^ Furthermore, evidence in long COVID patients show impaired peripheral-blood-mononuclear cell (PBMC) respiration and reduced spare respiratory capacity, together with abnormal muscle and brain high-energy phosphate dynamics on MR spectroscopy, and elevated circulating markers of oxidative stress and mitochondrial damage.^22^^,^^23^ These changes plausibly contribute to core symptoms such as fatigue, post-exertional malaise and cognitive slowing through sustained ATP deficit and ROS-driven activation of NF-κB and NLRP3.^24^^,^^25^^,^^26^ Moreover, additional mechanistic evidence shows that the viral non-structural protein Nsp8 localizes to mitochondria, induces ROS-mediated mitochondrial injury, and triggers incomplete mitophagy.^13^ In summary, the direct viral attack on mitochondria and indirect infection consequences like, pneumonia caused hypoxic environment and increased ROS amount due to neutrophil responses, converge on mitochondrial dysfunction during acute phase, but the repair phase involves mitophagy, mitochondrial fission/fusion remodeling, disturbed heme metabolism (Figure 2G).

Tryptophan-kynurenine pathway dysregulation reflects systemic immunometabolic stress. In our cohort, urinary kynurenine was significantly elevated during the acute phase, although tryptophan-kynurenine alterations were not sustained at three months at the group level, we highlight this pathway because growing evidence suggests that even transient or early dysregulation may have lasting functional consequences in susceptible individuals.^27^ Furthermore, mechanistic studies demonstrate prolonged activation of the kynurenine pathway via IDO2 induction, mitochondrial impairment, and accumulation of neuroactive downstream metabolites (quinolinic acid, kynurenic acid) in PASC patients.^28^ Beyond neuromodulation, this pathway provides a *de novo* source of NAD^+^, linking it to mitochondrial redox balance and cellular energy homeostasis. This has therapeutic implications, as modulation of the kynurenine pathway, NAD^+^-restoring interventions aimed at reducing downstream neurotoxic kynurenines have been proposed in myalgic encephalomyelitis/ chronic fatigue syndrome (ME/CFS).^29^^,^^30^ Interestingly, in our integrative correlation analysis between kidney damage biomarkers and urine metabolites collagen IV strongly correlated with kynurenine, tryptophan, and other amino acids, possibly linking basement membrane integrity to systemic immunometabolism**.**

### Limitations of the study

Several limitations should be considered when interpreting these findings. First, the EHR-based stratification of long COVID and recovered groups depends on routine clinical coding, which enables scalable identification of post-acute sequelae but may introduce misclassification due to variability in documentation practices by clinicians. Second, the cohort consists of hospitalized patients with diverse pre-COVID comorbidities, contributing biological heterogeneity that may obscure subtler disease-specific signals and limit power for phenotype group analysis (long COVID vs. recovered). Hospitalized individuals also frequently exhibit multi-organ involvement, complicating attribution of blood-derived molecular signatures to discrete pathophysiological processes. Third, long COVID itself is clinically heterogeneous, with multiple, potentially distinct mechanisms represented within the same group, making unified molecular interpretation challenging. Finally, although our multi-omics design performed blood transcriptomics, targeted metabolomics, and kidney injury biomarkers, it cannot capture tissue-specific processes. Also, we note that our study is observational and based on bulk blood transcriptomics; therefore, the identified immunothrombosis signatures should be interpreted as molecular associations rather than direct evidence of causality or tissue-specific endothelial activation. Adding to that, bulk blood RNA-seq lacks cellular resolution, and the targeted metabolite panel provides only partial metabolic coverage.

## Resource availability

### Lead contact

Further information and requests for resources should be directed to and will be fulfilled by the lead contact, Laura Ansone (laura.ansone@biomed.lu.lv).

### Materials availability

This study did not generate new unique reagents.

### Data and code availability

• The transcriptome dataset generated during this study is available in the European Genome-phenome Archive (EGA) under accession number EGAD50000002067, https://ega-archive.org/datasets/EGAD50000002067. Access to controlled data are subject to approval by the relevant data access committee in accordance with ethical and regulatory requirements.
• This study does not report any original code.
• Any additional information required to reanalyze the data reported in this paper is available from the lead contact upon request.

## Acknowledgments

This research was supported within the framework of the European Union’s Recovery and Resilience Mechanism project No.5.2.1.1.i.0/2/24/I/CFLA/001 “Consolidation of the Latvian Institute of Organic Synthesis and the Latvian Biomedical Research and Study Center.”

The authors acknowledge the Latvian Biomedical Research and Study Center and the Genome Database of the Latvian Population for the organization of participants’ recruitment and for providing the infrastructure for sample processing and storing, and data storage.

## Author contributions

L.A., M.B., and J.K. conceptualized and designed the study, and M.B. and J.K. supervised the project. L.A., L.P., K. Kornejevs, and D.B. performed RNA extraction and sample processing, while L.P., K. Kornejevs, and D.B. prepared transcriptome libraries and K.M. and L.E. performed sequencing. L.A. and L.P. conducted urine sample preparation and biomarker and metabolomics assays, and A.V. and K. Klavins performed metabolomics data acquisition and analysis. V.R. supervised patient recruitment and clinical and EHR data acquisition. R.S. and I.S. performed bioinformatics analysis, and L.A. performed statistical analysis. L.A., M.B., J.K., and H.B.S. interpreted the data. L.A. wrote the original draft, and H.B.S. critically revised the manuscript. All authors reviewed and approved the final manuscript. L.A. coordinated the study, performed data analysis, and serves as the primary contact with the journal, while J.K. supervised the study and provided senior oversight.

## Declaration of interests

The authors declare no competing interests.

## Declaration of generative AI and AI-assisted technologies in the writing process

During the preparation of this work, the author(s) used ChatGPT to improve the clarity and readability of the manuscript by refining phrasing and English-language quality, as English is not the authors’ first language, and to assist with condensing the wording of methodological descriptions without altering their scientific content. After using this tool, the author(s) reviewed and edited the content as needed and take(s) full responsibility for the content of the published article.

## STAR★Methods

### Key resources table

**Table:** 

REAGENT or RESOURCE	SOURCE	IDENTIFIER
**Biological samples**		

Whole blood samples from hospitalized COVID-19 patients (*n* = 81)	Genome Database of the Latvian Population	This study
Urine samples from hospitalized COVID-19 patients (*n* = 61)	Genome Database of the Latvian Population	This study

**Chemicals, peptides, and recombinant proteins**		

Methanol (LC-MS grade)	Sigma-Aldrich (Merck)	Cat# 34966
Acetonitrile (LC-MS grade)	Sigma-Aldrich (Merck)	Cat# 34998
Formic acid	Sigma-Aldrich (Merck)	Cat# 56302
Ammonium formate	Sigma-Aldrich (Merck)	Cat# 70221

**Critical commercial assays**		

Tempus™ Blood RNA Tubes	Thermo Fisher Scientific	Cat# 4342792
Tempus™ Spin RNA Isolation Kit	Thermo Fisher Scientific	Cat# 4380204
TURBO DNA-free™ Kit	Thermo Fisher Scientific	Cat# AM1907
MGIEasy rRNA Depletion Kit	MGI Tech	Cat# 1000005955
MGIEasy RNA Directional Library Prep Kit	MGI Tech	Cat# 1000006386
DNBSEQ-G400RS High-throughput Sequencing Set (FCL PE150)	MGI Tech	Cat# 1000016954
Metabolomics Amino Acid Mix Standard	Cambridge Isotope Laboratories	Cat# MSK-A2-1.2
Carnitine Standards Set B	Cambridge Isotope Laboratories	Cat# NSK-B-1
MILLIPLEX® Human Kidney Injury Magnetic Bead Panel 1	MilliporeSigma (Merck)	Cat# HKI1MAG-99K
MILLIPLEX® Human Kidney Injury Magnetic Bead Panel 2	MilliporeSigma (Merck)	Cat# HKI2MAG-99K

**Deposited data**		

RNA-seq raw data	European Genome-phenome Archive (EGA)	EGAD50000002067

**Software and algorithms**		

FastQC	https://www.bioinformatics.babraham.ac.uk/projects/fastqc/	N/A
Cutadapt v1.16	https://cutadapt.readthedocs.io	Martin^31^
Trimmomatic v0.39	http://www.usadellab.org/cms/?page=trimmomatic	Bolger et al.^32^
STAR aligner v2.5.3a	https://github.com/alexdobin/STAR	Dobin et al.^33^
R (v4.x)	https://www.r-project.org/	N/A
Limma	https://bioconductor.org/packages/limma/	Ritchie et al.^34^
edgeR	https://bioconductor.org/packages/edgeR/	Robinson et al.^35^
sva (ComBat)	https://bioconductor.org/packages/sva/	Leek et al.^36^
mixOmics	http://mixomics.org/	Rohart^37^
Mfuzz	https://bioconductor.org/packages/Mfuzz/	Kumar & Futschik^38^
clusterProfiler	https://bioconductor.org/packages/clusterProfiler/	Yu^39^
TraceFinder 5.2	Thermo Fisher Scientific	TRACEFINDER5

### Experimental model and study participant details

The patients were admitted in Latvian Center of Infectious Diseases, Riga East University Hospital. All patients had confirmed SARS-CoV-2 infection by antibody or qPCR testing at the time of hospital admission. Written informed consent was obtained from every participant before inclusion in the study, and the study protocol was approved by the Central Medical Ethics Committee of Latvia (No. 01-29.1.2/928). All patients were enrolled in Latvian national biobank.^40^

The acute phase samples were collected upon admission (Timepoint A): blood samples on the first day of admission and urine samples on the second day after admission. Collection of longitudinal samples (Timepoints B and C) was organized in an ambulatory setting, where patients provided pre-collected urine samples and underwent blood collection in a certified clinical laboratory (E. Gulbja Laboratorija Ltd., Riga, Latvia). All samples were collected during the same period (2020–2021).

Patient sex and age were recorded for all participants and are reported in Table 1. Sex was included as a covariate in all statistical models. No stratified analysis by sex was performed. None of the patients were vaccinated with any of the COVID-19 vaccines before hospitalization or during the sample collection period.

During hospitalization, patients received standard-of-care supportive therapy including supplemental oxygen (typically 2–4 L/min), anticoagulation (predominantly low–molecular weight heparin, with occasional direct oral anticoagulant use), and systemic corticosteroids (most commonly dexamethasone). In addition, many patients received symptomatic treatments (e.g., antipyretics/analgesics, mucolytics, proton-pump inhibitors) and empiric antibiotics, while antiviral treatment was used in a subset of patients depending on the treatment period (including remdesivir in later cases).

Hematological and biochemical analyses in the acute phase were performed at the hospital’s clinical lab but during recovery in a certified clinical laboratory (E. Gulbja Laboratorija, Ltd., Riga, Latvia).

Health registry data were obtained for all patients, and participants were retrospectively stratified into two phenotype groups (Long COVID and Recovered). Long COVID patients were defined as individuals who developed a new post-acute sequelae of SARS-CoV-2 infection (PASC) diagnosis within 12 months following the acute COVID-19 phase that had not been present during the three-year pre-COVID background period. Recovered patients did not receive new PASC-related diagnoses during the same follow-up period. PASC diagnosis selection from the published work of Zang et al.^5^

No cell lines or primary cell cultures were used in this study.

#### Ethics statement

The study was carried out in accordance with the principles of the Declaration of Helsinki, and the study protocol was approved by the Central Medical Ethics Committee of Latvia (No. 01-29.1.2/928). Informed consent was obtained from all participants at the beginning of the study.

### Method details

#### Blood, and urine sample collection, RNA extraction

The sample collection from COVID-19 patients was conducted by medical personnel according to the study design. Venous blood samples (3 mL) from participants were collected into *Tempus™ Blood RNA tubes (6 mL),* which contain an RNA-stabilizing reagent that lyses cells and inactivates Rnases. Following sample collection, the tubes were transported to LBMC and stored at −80°C until RNA extraction. RNA extraction was performed using the *Tempus™ Spin RNA Isolation Kit (Thermo Fischer Scientific)*, which employs a column chromatography-based method. *TURBO DNA-free Kit (Thermo Fischer Scientific)* was also used to degrade any possible DNA present in the sample, the extracted RNA was stored at −80°C until sequencing library preparation. The patients were instructed to collect first urine in the morning, middle flow, in a sterile plastic tube and stored at 4 °C for maximum 5 h before centrifugation (2500xg) for 10 min. Supernatant was then stored at 80 °C in aliquots by 2 mL until processing for metabolomics or biomarker analysis.

#### Transcriptome sequencing

The *MGIEasy rRNA Depletion Kit (MGI tech)* was used to purify mRNA by removing ribosomal RNAs. After hybridizing the sample with the probes, the targeted RNAs were removed using magnetic RNA cleanup beads.The *MGIEasy RNA Directional Library Prep Kit (MGI tech)* was used to prepare cDNA libraries by synthesizing complementary DNA (cDNA) from RNA in the samples. This process employed reverse transcriptase, an RNA-dependent DNA polymerase, which catalyzes the synthesis of a DNA strand using RNA as a template. The *MGIEasy RNA Directional Library Prep Kit (MGI tech)* was used to prepare cDNA libraries by synthesizing complementary DNA (cDNA) from RNA in the samples. Then, libraries were sequenced using the *DNBSEQ-G400RS High-throughput Sequencing Set (FCL PE150*) *(MGI tech)* reagent kit on the *DNBSEQ-G400* sequencing platform.

#### Targeted metabolomics with HILIC-MS

Urine samples were thawed on ice at 4 °C for 30–60 min and clarified by mild centrifugation at 2,000 g for 10 min at 4 °C. Internal standard (ISTD) mix was prepared by diluting the *Metabolomics Amino Acid Mix Standard (Cambridge Isotope Laboratories, MSK-A2-1.(2)* 1:10 in H_2_O, dissolving the labeled *Carnitine Standards Set B (Cambridge Isotope Laboratories, NSK-B-(1)* in 1 mL H_2_O, and combining 100 μL of the diluted amino acid ISTD with 250 μL of the prepared carnitine ISTD solution, followed by addition of 650 μL H_2_O. For sample extraction, 10 μL of urine was mixed with 80 μL methanol and 10 μL of the ISTD solution, vortexed twice for 15 s, and centrifuged at 10,000 rpm for 10 min at 4 °C. The resulting supernatant was carefully transferred to HPLC vials, and samples were subjected to LC-MS analysis.

Targeted quantitative metabolite analysis was conducted using HILIC-MS- OrbitrapExploris 120 system *(Thermo Fischer Scientific)*, ACQUITY UPLC BEH Amide 1.7 μm 2.1 × 100 mm analytical column by Waters was used for metabolite separation, employing a gradient elution of 0.15%formic acid and 10 mM ammonium formate in water as mobile phase A, and a solution of 0.15% formic acid and 10 mM ammonium formate in 85% acetonitrile as mobile phase B. The Orbitrap Exploris 120 *(Thermo Fisher Scientific)* was utilized for MS detection, operated in electrospray ionization (ESI) positive mode. Full-scan acquisition was performed over an m/z range of 50–400 at a resolution of 30,000. The ESI spray voltage was set to 3.5 kV in positive mode. The ion transfer tube temperature was 350 °C, and the vaporizer temperature was 400 °C. Sheath gas and auxiliary gas flow rates were set to 50 and 12 arbitrary units, respectively.

#### Kidney biomarker multiplexing with luminex 200

Kidney biomarker multiplexing was performed using Luminex 200*® (Thermo Fisher Scientific),* a dual-laser flow detection instrument. The samples were prepared using *MILLIPLEX® Human Kidney Injury Magnetic Bead Panel 1 - Toxicity Multiplex Assay (Merck Millipore)* and *MILLIPLEX® Human Kidney Injury Magnetic Bead Panel 2 - Toxicity Multiplex Assay (Merck Millipore)* reagent kits, where beads are coated with specific antibodies and different concentrations of two fluorescent dyes in order for these beads to bind to specific analytes from the sample and emit certain color combinations. These analytes were Epidermal growth factor (EGF), Lipocalin 2, Albumin, Cystatin C, Osteopontin (OPN), α-1-Microglobulin, Glutathione S-transferase alpha (GSTα), Tissue inhibitor of metalloproteinase-1 (TIMP-1), Kidney Injury Molecule-1 (KIM-1), Interferon gamma-induced protein 10 (IP-10), Renin, Fatty Acid Binding Protein 1 (FABP1), Collagen IV and Trefoil factor 3 (TFF-3). After the results were read by the Luminex 200 instrument, the computer program xPONENT automatically quantified the concentrations of each analyte in ng/mL.

### Quantification and statistical analysis

Statistical details, including exact sample sizes (n), statistical tests, and adjusted *p*-values, are provided in the results, figure legends, and supplemental tables.

#### Transcriptome data analysis

##### Bioinformatical analysis

Quality control of sequencing reads was performed using *FastQC*. Adapter trimming was conducted with *cutadapt v1.16*, while read trimming at the 5′ and 3′ ends was carried out using *Trimmomatic v0.39* with the following settings: window size of 5, quality threshold of 20, and a minimum read length of 75 bp for subsequent analysis. Reads were aligned to the human reference genome *GRCh38 release 90*, and transcript-level read counts were calculated using the *STAR 2.5.3a aligner.* ***Normalization and Batch Correction***. Gene expression analysis was performed using the limma-edgeR pipeline in R. Low-expression genes were filtered using a threshold of CPM >1 in at least n samples (where *n* = 1/3 of the smallest group size). TMM normalization was applied using calcNormFactors(). Batch effects were corrected using *ComBat* from the sva package, preserving biological variables of interest (Group, Timepoint, Sex) in the design matrix. ***Statistical analysis.*** *Longitudinal design.* Statistical model: linear mixed-effects modeling was implemented using limma’s *duplicateCorrelatio*n function to account for repeated measures within subjects. The design matrix was specified as ∼0 + Timepoint + Sex, with Subject as a blocking factor. Within-subject correlation was estimated using *duplicateCorrelation()* and incorporated into subsequent analyses using *lmFit()* with block and correlation parameters. *Cross-sectional design analysis***.** Group differences at each timepoint were assessed using separate limma analyses. For each timepoint (A, B, C), a design matrix was specified as ∼0 + Group + Sex, utilizing the batch-corrected expression data. Linear modeling was performed using lmFit() with trend = TRUE for log-CPM data. ***Sparse Canonical Correlation Analysis (sCCA).*** sCCA was performed specifically on Long COVID patients at timepoint C (*n* = (46) to identify gene-symptom associations. Symptom categories were derived from EHR ICD-10 diagnostic codes and converted to binary variables: Respiratory, General/Musculoskeletal, Nervous, Circulatory, Genitourinary, Digestive, and Endocrine, ICD-10 codes in each category can be found in Table 1 sCCA was implemented using the *mixOmics* package (v6.x) with the spls() function in canonical mode. Analysis parameters: ncomp = 3 components, keepX = c(30, 25, 20) genes per component, keepY = c(5, 4, (3) symptoms per component.

##### Fuzzy clustering of gene trajectories with mfuzz

Fuzzy c-means clustering of longitudinal gene expression trajectories was performed using the *Mfuzz* package (v2.x) in R to identify distinct temporal patterns during COVID-19 recovery. A targeted gene set was constructed using a two-tier approach: Tier 1 comprised genes significantly differentially expressed between Long COVID and Recovered groups at timepoint C (FDR <0.05), while Tier 2 included the top 500 genes from longitudinal analysis (timepoint A vs. C) ranked by hybrid score (-log_10_(FDR) × |log_2_FC|), excluding genes already present in Tier 1. Expression data were filtered to include only subjects with complete longitudinal profiles across all three timepoints (A, B, C), resulting in 62 subjects (39 Long COVID, 23 Recovered). Gene expression matrices were created by averaging expression values across timepoints for each group separately, generating subject-averaged temporal profiles. Prior to clustering, expression data were standardized using the standardise() function to ensure equal weighting across genes. Optimal fuzziness parameters (m) were estimated independently for each group using the mestimate() function, which determines the appropriate degree of membership overlap between clusters. Fuzzy c-means clustering was performed with k = 4 clusters using the mfuzz() function, with cluster membership values ranging from 0 to 1 for each gene-cluster pair. Genes were assigned to clusters based on maximum membership values, and cluster quality was assessed by mean membership strength across all gene assignments. *Pathway enrichment analysis* was conducted for each cluster using genes with membership >0.5, testing against Gene Ontology Biological Process, KEGG, Reactome, and Hallmark gene sets via clusterProfiler (v4.x) with Benjamini-Hochberg FDR correction (q < 0.05). This approach enabled identification of distinct molecular programs underlying different recovery trajectories in Long COVID versus Recovered patient groups.

#### Targeted metabolomics data analysis

Acquired raw LC-MS data were processed and analyzed using TraceFinder 5.2 software. A seven-point linear calibration curve with internal standardization and 1/x weighing was constructed to quantify of the metabolites. A total of 46 metabolites were quantified and analyzed. Missing values were imputed using 1/5 of the minimum positive value for each metabolite to handle values below the limit of detection. Data underwent log2 transformation with addition of a small constant (minimum positive value/10) to handle zero values, followed by Pareto scaling (mean-centered and divided by square root of standard deviation) to reduce the influence of high-abundance metabolites while preserving data structure. *Longitudinal Analysis.* Longitudinal changes were analyzed using limma with a linear mixed-effects approach. The design matrix was specified as ∼Timepoint + Batch + Sex, with Subject as a blocking factor using *duplicateCorrelation()* to account for repeated measures. Within-subject correlation was estimated and incorporated into the model using *lmFit()* with block and correlation parameters. Three pairwise timepoint comparisons were performed: (1) A vs. B, (2) A vs. C, and (3) B vs. C, using makeContrasts() followed by empirical Bayes moderation with eBayes(). *Cross-sectional Analysis.* Group differences at each timepoint were assessed using separate limma analyses with design matrix ∼0 + Group + Batch + Sex. The contrast Long COVID vs. Recovered was tested at each timepoint using lmFit() and contrasts.fit().

#### Kidney biomarkers in urine by LUMINEX, biomarker and metabolite association

Biomarker concentrations were preprocessed prior to statistical analysis. Values below the limit of detection were conservatively estimated as one-tenth of the minimum observed positive value. Biomarker data were log_2_-transformed and subsequently *Z* score normalized to enable cross-marker comparison. Longitudinal and cross-sectional analyses were performed using the limma framework with linear mixed-effects modeling, incorporating subject ID as a blocking factor to account for repeated measures and adjusting for batch effects and sex.

To explore relationships between renal injury and systemic metabolism, correlation analysis was performed between urinary kidney biomarkers and targeted urinary metabolites measured by LC-MS. Biomarker and metabolite datasets were first harmonized by matching samples based on subject identifier and timepoint. Only samples present in both datasets were retained. To balance statistical power and data completeness, samples with excessive missingness (>50% missing biomarkers or >30% missing metabolites) were excluded; remaining missing values were imputed using median imputation. Spearman rank correlation coefficients were calculated for all pairwise biomarker–metabolite combinations. Resulting *p*-values were adjusted for multiple testing using the Benjamini–Hochberg procedure. Associations with FDR <0.05 were considered statistically significant, while FDR <0.10 was additionally reported for exploratory network analyses. Correlation networks were constructed by retaining only strong associations (|r| > 0.5, FDR <0.05), visualized as bipartite networks separating biomarkers and metabolites into distinct node classes. Edge color indicated correlation direction, and edge thickness reflected effect size. This integrative analysis framework and network construction are detailed in the biomarker–metabolite correlation script.

## Supplemental information

Table S1. Summary of clinical and demographic characteristics of the study cohort

Table S2. Differential expression and pathway analyses across time points Table S2A. Differentially expressed genes between time points A and B. Table S2B. Differentially expressed genes between time points B and C. Table S2C. Differentially abundant metabolites between time points A and B. Table S2D. Kidney injury biomarker analysis. Table S2E. MFuzz clustering results. Table S2F. KEGG pathway enrichment analysis. Table S2G. Reactome pathway enrichment analysis.

Table S3. Multi-omics differences between Long COVID and recovered patients Table S3A. Differential analysis between Long COVID and recovered patients. Table S3B. Additional differential analysis between Long COVID and recovered patients
